# Calcifediol or Corticosteroids in the Treatment of COVID-19: An Observational Study

**DOI:** 10.3390/nu16121910

**Published:** 2024-06-17

**Authors:** Marta Entrenas-Castillo, Luis Manuel Entrenas-Costa, María P. Pata, Bernabe Jurado-Gamez, Cristina Muñoz-Corroto, Cristina Gomez-Rebollo, Estefania Mira-Padilla, Roger Bouillon, Jose Manuel Quesada-Gómez

**Affiliations:** 1 Hospital QuironSalud Córdoba, 14004 Córdoba, Spain; marenca@gmail.com (M.E.-C.); lmentrenas@uco.es (L.M.E.-C.); 2 Instituto Maimónides de Investigación Biomédica de Córdoba (IMIBIC), Universidad de Córdoba, 14004 Córdoba, Spain; bjg01co@hotmail.com; 3 Unidad de Gestión Clínica de Neumología, Hospital Universitario Reina Sofía, 14004 Córdoba, Spain; cmcorroto@gmail.com (C.M.-C.); crisgrebollo@gmail.com (C.G.-R.); estefaniamirapadilla@gmail.com (E.M.-P.); 4 Biostatech, 15782 Santiago de Compostela, Spain; mariapata6@biostatech.com; 5 Laboratory of Clinical and Experimental Endocrinology, Department of Chronic Diseases, Metabolism and Ageing, KU Leuven, 3000 Leuven, Belgium; 6 Unidad de Gestión Clínica de Endocrinología y Nutrición, Hospital Universitario Reina Sofía, 14004 Córdoba, Spain; 7 CIBER de Fragilidad y Envejecimiento Saludable (CIBERFES), 28029 Madrid, Spain; 8 Departamento de Enfermería, Farmacología y Fisioterapia, Universidad de Córdoba, 14004 Córdoba, Spain

**Keywords:** COVID-19, corticoids, calcifediol, SARS-CoV-2, COVID-19 drug treatment, vitamin D

## Abstract

Medical treatment of coronavirus 19 disease (COVID-19) is a therapeutic challenge. The available data strongly suggest that calcifediol treatment may reduce the severity of COVID-19, and corticosteroids are the treatment of choice worldwide for severe COVID-19. Both have a very similar action profile, and their combined use in patients may modify the contribution of each administered compound. Objective: To evaluate how treatment with calcifediol and/or corticosteroids in medical practice modified the need for ICU admission, death, or poor prognosis of patients hospitalized with COVID-19 during the first outbreaks. Design, patients and setting: A retrospective observational cohort study of patients admitted for COVID-19 to the Pneumology Unit of the Hospital Universitario Reina Sofía (Córdoba, Spain). Interventions: Patients were treated with calcifediol or/and corticosteroids with the best available therapy and standard care, according to clinical practice guidelines. Measurements: Admission to the intensive care unit (ICU) or death during hospitalization and poor prognosis. Results: Seven hundred and twenty-eight patients were included. According to the treatment received, they were included in four groups: calcifediol (*n* = 68), glucocorticoids (*n* = 112), both (*n* = 510), or neither (*n* = 38). Of the 578 patients treated with calcifediol, 88 were admitted to the ICU (15%), while of the 150 not treated with calcifediol, 39 required ICU admission (26%) (*p* < 0.01). Among the patients taking calcifediol without glucocorticoids, only 4 of 68 (5.8%) required ICU admission, compared to 84 of 510 (16.5%) treated with both (*p* = 0.022). Of the 595 patients who had a good prognosis, 568 (82.01%) had received treatment with calcifediol versus the 133 patients with a poor prognosis, of whom 90 (67.66%) had received calcifediol (*p* < 0.001). This difference was not found for corticosteroids. Interpretation: The treatment of choice for hospitalized patients with moderate or mild COVID-19 could be calcifediol, not administering corticosteroids, until the natural history of the disease reaches a stage of hyperinflammation.

## 1. Introduction

Coronavirus 19 disease (COVID-19), caused by the β-coronavirus SARS-CoV-2, represents the greatest challenge to modern medicine and public health systems worldwide [1]. At the beginning of the pandemic, there were no specific antiviral treatments and no proven treatments for the disease, and 20% of patients developed severe symptomatology, while 5% developed acute respiratory distress syndrome (ARDS), septic shock and multi-organ failure, accompanied by a high risk of death [2,3].

The natural history of COVID-19 and the severity of the course of SARS-CoV-2 infection are conditioned by cell tropism and the host immune response. The severity of the disease is related to a highly dysregulated innate immune response, which is generally characterized by a delayed, diminished or absent interferon I response induced by the virus, which is related to the onset of symptoms [4]. This facilitates maximal virus replication and shedding (which may be implicated in the tissue persistence of the virus described in some patients). This is followed by a potent inflammatory response, with exuberant production of inflammatory cytokines and chemokines in the so-called cytokine storm [5,6,7] and activation of the renin–angiotensin–aldosterone system (RAAS), with decreased angiotensin-converting enzyme (ACE2) [8], inappropriate recruitment of inflammatory populations of monocytes and macrophages and tissue damage, starting in, and predominantly affecting, the lungs, leading to ARDS, with multiple extrapulmonary manifestations [9]. In addition, the hyperinflammatory state and RAAS activation are intimately involved in altering the coagulation cascade, which, in cooperation with endothelial cell infection and endothelitis, leads to the prothrombotic state observed in ARDS during SARS-CoV-2 infections [10].

The severity of the process and the urgent need for treatment led researchers to develop at an astonishing pace effective vaccines to provide immune protection [2], antiviral agents, anti-inflammatory agents, anti-SARS-CoV-2 antibodies, treatments for acute hypoxemic respiratory failure, antithrombotics and renin–angiotensin–aldosterone system modulators against the disease [11], as well as using the strategy of repositioning safe drugs, for which pharmacokinetic and toxicity information was available, approved for another indication and repurposed to improve the symptoms and clinical outcomes in patients with COVID-19, reducing disease burden, hospital admissions, deaths and long-term sequelae.

Numerous drugs have been investigated with this strategy [12], and some have been successfully tested. Calcifediol (25-hydroxyvitamin D3;25OHD3), a cornerstone and prohormone of the vitamin D endocrine system (VDES), was one of them. It requires only one hydroxylation to become 1,25(OH)2D3 (calcitriol), the hormone of the VDES that exerts its functions by activating the vitamin D receptor (VDR), which is expressed in the immune system and many other affected organs in COVID-19, including the lungs, intestine and cardiovascular system [13]. The available data strongly suggest that treatment with calcifediol can decrease the severity of COVID-19, as shown by the decreased need for intensive care unit (ICU) admissions and by decreased risk of death [14,15,16].

Corticosteroids have been commonly used for their immunosuppressive/anti-inflammatory properties in inhibiting the expression of multiple pro-inflammatory cytokines/chemokines and the (in)activation of various immune cells [17], but at the start of the pandemic, corticosteroid treatment in COVID-19 was formally contraindicated [18]. For this reason, the initial studies that treated COVID-19 successfully with calcifediol and the best available therapy did not use corticosteroids [14,15,16], except in exceptional cases and after a well-motivated and consensual clinical decision. Thus, the results obtained did not account for the confounding factor of the modulation of innate and acquired immunity by corticosteroid treatment [17].

Following the RECOVERY trial [19], corticosteroids have become the paradigm of success as a repositioning and reference treatment in the management of COVID-19, changing clinical practice guidelines worldwide through recommendation of their use in hospitalized patients with severe COVID-19, requiring intensive oxygen therapy, to mitigate the development of cytokine/chemokine storms and minimize ARDS and severe multi-organ damage [20]. However, corticosteroid therapy may compromise viral clearance and increase mortality in patients treated in the early stages of disease [21,22]. Moreover, this treatment may not be safe due to interference with coagulation and metabolic pathways and it potentially increasing the risk of uncommon infections [23].

Corticosteroids and the VDES have a very similar action profile, and their combined use in patients with COVID-19 may modify the contribution of each of them individually [24].

Nevertheless, other drugs repositioned for use in the disease, such as hydroxychloroquine and/or azithromycin, were withdrawn from use due to their lack of efficacy and adverse effects [25].

The aim of this observational study is to evaluate how treatment with calcifediol and/or corticosteroids in real-world medical practice modified the risk of ICU admission, death, or the prognosis of patients hospitalized with COVID-19 during the first outbreaks of the pandemic before the first vaccinations.

## 2. Patients and Methods

### 2.1. Overview of the Study

This retrospective observational cohort study was approved by the Biomedical Research Ethics Committee of the Hospital Universitario Reina Sofía (committee reference number 5291), registered in the ClinicalTrials.gov public database (NCT05819918), which has an accessible and detailed description of the study protocol.

The patients, admitted for COVID-19 to the Pneumology Unit of the Hospital Universitario Reina Sofía (Córdoba, Spain), had to meet all the inclusion criteria: (1) age ≥ 18 and ≤90 years; (2) interstitial pneumonia characterized by the presence of infiltrates on chest X-ray or CT scan; (3) SARS-CoV-2 infection, confirmed by a positive antigen detection test or polymerase chain reaction assay; (4) CURB 65 scale > 1, and the exclusion criteria included not being able to retrieve this information from electronic medical records. They were assigned, according to their date of admission, to one of the three epidemic waves, following the distribution of the Spanish National Epidemiological Surveillance Network: first wave (from the beginning of the pandemic to 21 June 2020, the date on which the state of alarm in Spain ended); second wave (from 22 June to 6 December 2020, the 14-day cumulative incidence [AI] turning point); third wave from 7 December 2020 to 14 March 2021 (14-day AI turning point). Patients hospitalized from 10 March 2020 to 4 March 2022 were included. The patients were followed up during admission until discharge and at 30, 60 and 90 days telemetrically.

### 2.2. Statistical Methods

A descriptive analysis was performed for all variables. In order to classify the prognosis of each patient, a composite variable was generated, defining poor prognosis as the need for admission to the intensive care unit (ICU) or death during hospitalization and good prognosis otherwise. A bivariate association analysis was performed between the variables and the groups of patients classified according to treatment with calcifediol and corticoids. Four groups of patients were generated: those treated with calcifediol alone; with corticosteroids alone, according to the protocol of the Hospital Universitario Reina Sofía; with both; with neither. Bivariate association for categorical variables was tested through the chi-square test or Fisher’s exact test, while the association for quantitative variables was tested through the Kruskal–Wallis test or the Mann–Whitney test. For those variables that were statistically significant, a post hoc test was applied (Fisher’s test or Dunnett’s test for categorical or quantitative variables, respectively). The *p*-values were adjusted using Bonferroni correction.

Odds ratios (ORs) and 95% confidence intervals were estimated for the risk of ICU admission, mortality and prognosis in relation to the timing of calcifediol administration relative to corticosteroid administration.

The R program, version 4.2.1 (R Core Team, 2022), was used for all calculations. An alpha level of 0.05 was established for statistical testing. The Strengthening the Reporting of Observational Studies in Epidemiology (STROBE) checklist was followed in the preparation of this report.

## 3. Results

### 3.1. Characteristics of the Study Population

**Figure 1:**
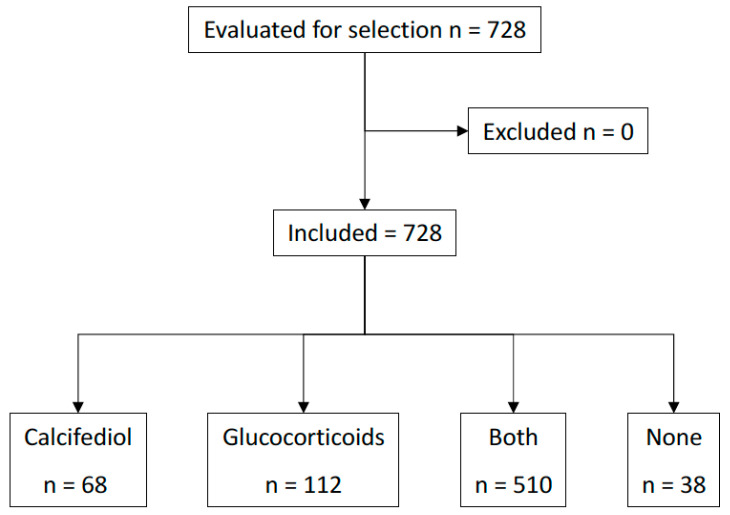
Patient flow diagram.

A total of 728 patients met all the inclusion criteria and none of the exclusion criteria. A flow diagram for the study’s inclusion and exclusion is provided in Figure 1. Table 1 summarizes the demographics of the population by treatment group. A total of 127 patients (17%) were admitted to the ICU, and 25 (3%) died. In the 90-day follow-up period after discharge, 31 patients (4%) required readmission, with 1 patient dying before 60 days after hospital discharge.

The analysis focused on studying the effect of calcifediol and corticosteroids on avoiding admission to the ICU, death and poor prognosis. To this end, the patients were divided into four groups, according to the treatment received: treated with calcifediol (*n* = 68), with glucocorticoids (*n* = 112), with both (*n* = 510) or with neither (*n* = 38). We found no significant differences in the gender or age distribution between the four groups. Table 2 summarizes the personal history by treatment group. Table 3 summarizes the values of the analytical variables by group that showed significance.

During admission, patients could receive, in addition to corticosteroids or calcifediol, the “best available therapy”, agreed by a multidisciplinary team following the recommendations issued by the Ministry of Health [26]. Table 4 summarizes the drugs administered and the number of patients who received them by treatment group.

In terms of their personal history, the patients who received calcifediol had a significantly higher percentage of obesity/overweight than those who did not (82.3% vs. 16.7%, *p* < 0.01). But the other risk factors for severity of the infection were not significantly different.

The treatment distribution was not homogeneous. In wave 1, the use of calcifediol alone predominated (59.4%), while waves 2 and 3 were characterized by using calcifediol and glucocorticoids (71.3% and 93.6%, respectively) (*p* < 0.001). Also, the percentage of patients receiving calcifediol varied significantly (*p* < 0.001) throughout the three waves, reaching 95.1% of patients in the third wave.

The percentage of patients who received corticosteroids also increased significantly (*p* < 0.001) throughout the different waves. Among corticosteroids, dexamethasone was the treatment of choice, but high or pulse doses (1 mg methylprednisolone or equivalent per kilogram of body weight) were also used. The personal history of patients who received glucocorticoids vs. those who did not only showed a difference in the case of obesity/overweight (97.7% with obesity/overweight, vs. 2.3 without; *p* < 0.001) (Table 2).

### 3.2. Intensive Care Unit Admission

**Figure 2:**
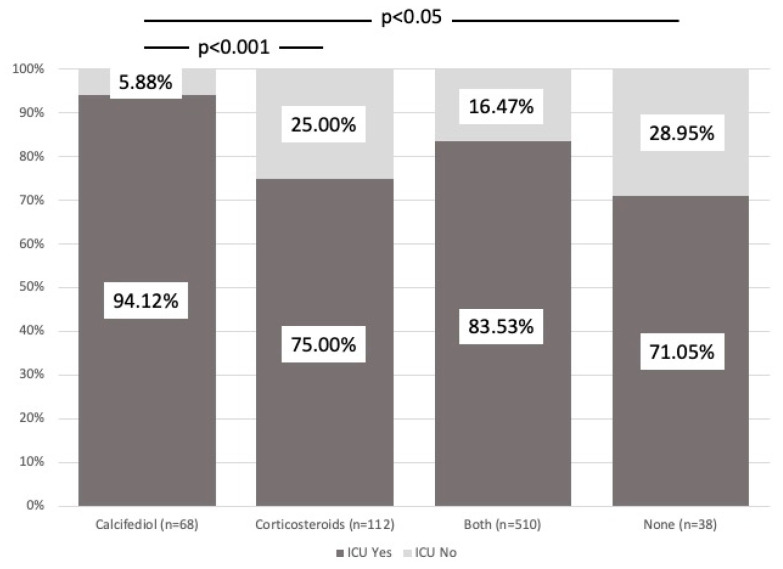
ICU admission. Comparison between groups. The figure shows the different percentages of each treatment group that required admission to the ICU (dark gray) or not (light gray). Patients who received calcifediol alone or in combination with corticosteroids showed a significantly lower percentage of need for ICU admission. χ^2^_Pearson_ = 14.58. *p* = 2.21 × 10^3^.

Figure 2 shows a comparison of the variable ICU admission rate for each of the four treatment groups. Of the 578 patients who received calcifediol, 88 were admitted to the ICU (15%), compared to 150 who did not take calcifediol, with 39 requiring admissions to the ICU (26%) (*p* < 0.01). However, among the patients taking calcifediol without glucocorticoids, only 4 of 68 (5.8%) required ICU admission, compared to 84 of 510 (16.5%) who received both treatments (*p* = 0.022).

Of the 622 patients who received glucocorticoids, 112 required ICU admission (18%), while of the 106 who did not take corticosteroids, 15 required ICU admission (14.1%) (*p* = 0.33). When the analysis evaluated patients taking corticosteroids without calcifediol, 28 of 112 patients (25%) required admission to the ICU.

The higher percentage of patients requiring admission to the ICU was significantly associated with the groups treated with corticosteroids alone (25% need for admission to the ICU) and those who did not receive any treatment (28.9% need for admission), while calcifediol showed a protective effect in helping avoid admission to the ICU (*p* < 0.01).

Of the total number of patients, 510 were treated with calcifediol and corticosteroids during hospitalization. A total of 67 of them received their first dose of calcifediol before their first dose of corticoids [B], 345 received both treatments simultaneously [S] and 98 received the first dose of calcifediol after the first dose of corticosteroids [A]. The timing of calcifediol administration relative to corticosteroid administration modified the risk of ICU admission (χ^2^, *p* < 0.001). Although all three therapeutic interventions (B-S-A) decreased the risk of ICU admission in relation to only corticoid use or no treatment, this risk was increased, regarding only calcifediol, when both drugs were given simultaneously, a total of 52 admissions out of 345 patients (15%) (OR: 2.74, 95% CI: 1.07–9.51) *p* < 0.05), and especially when calcifediol was given before (OR: 7.49, 95%CI: 2.63–27.7, *p* < 0.001), as opposed to when calcifediol was given after, corticosteroids, with 10 admissions out of 98 patients (10%) (OR: 1.77, 95%CI: 0.56–6.93).

### 3.3. Death

There was no significant association between death and treatment group, pandemic wave or sex, although the deceased patients were significantly older than the non-deceased (55.9 ± 11.5 vs. 52.6 ± 10.3 years) (*p* < 0.05). The patients who died had a significantly higher percentage of ICU admissions. Thus, of the 25 deceased patients, 19 (76.0%) were admitted to the ICU, while 6 (24.0%) were not (*p* < 0.001).

We analyzed the significance of the percentage of patients who died or not who only received calcifediol versus those who also received corticosteroids, here breaking down the order in which the former was prescribed, that is, B-S-A calcifediol. Although none of the three interventions showed statistical significance, giving calcifediol earlier was shown to be the favorable option for reducing the risk of death (OR 2.66 CI 95%: 0.63–24.9).

### 3.4. Poor Prognosis

**Figure 3:**
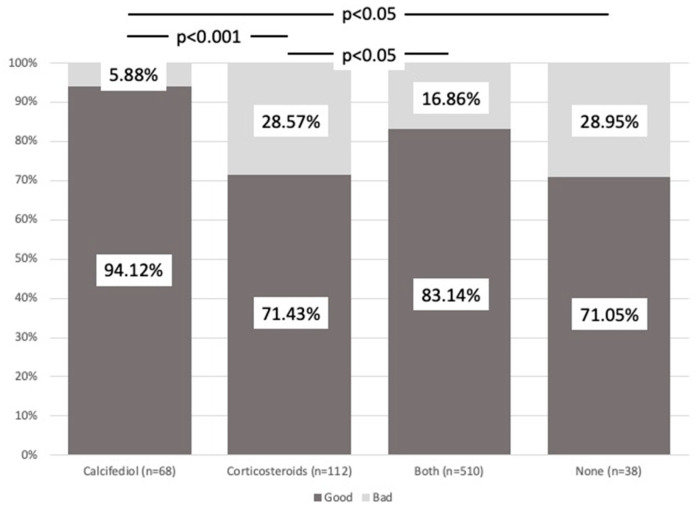
Prognosis. Comparison between groups. The figure shows the different percentages of each treatment group with poor (dark gray) or good (light gray) prognosis. Patients who received calcifediol alone or in combination with corticosteroids showed a significant percentage of better prognosis. χ^2^_Pearson_ = 18.53. *p* = 3.43 × 10^4^.

Poor prognosis differed between groups (χ^2^, *p* < 0.001), with the best outcome in those who received calcifediol (*p* < 0.001) (Figure 3).

Of the 595 patients who had a good prognosis, 568 (82.01%) had received treatment with calcifediol versus the 133 patients with a poor prognosis, of whom 90 (67.66%) had received calcifediol (*p* < 0.001). This difference was not found for corticosteroids.

We analyzed the significance of the percentage of patients with a good or poor prognosis who only received calcifediol versus those who, in addition, received glucocorticoids, breaking down the order in which these were prescribed, that is, before ([B]), simultaneously ([S]) or after ([A]) calcifediol. Although all three possibilities showed an association with a good prognosis ([B]: 44 of 67 patients, 49.25%; [A]: 88 of 98 patients, 89.79%; [S]: 292 of 345 patients, 84.63%) (*p* < 0.001), this was much more significant when both drugs were given simultaneously and especially when calcifediol was given before, while corticosteroids being given first was the least favorable option.

We assessed whether there was any difference in the prognosis of patients taking the two drugs and receiving corticosteroids before day 10 (from the first symptoms, due to the natural history of the disease) or before day 7 (according to the results of the RECOVERY trial). To perform these analyses, only patients taking both treatments were selected (*n* = 510). For each day from admission to corticosteroid administration, the risk of poor prognosis was significantly increased, by 22% [OR (95%CI) = 1.22 (1.08–1.37)].

We evaluated whether the administration of azithromycin, hydroxychloroquine or both added as the “best available therapy” improved prognosis compared to when using calcifediol alone. This analysis was performed in three groups of patients: first, all patients (*n* = 728); second, patients taking calcifediol alone plus patients taking neither corticosteroids nor calcifediol (*n* = 106); finally, patients taking calcifediol alone (*n* = 68).

The only significant protective factor was treatment with calcifediol, whereas azithromycin, azithromycin–hydroxychloroquine, calcifediol–azithromycin and hydroxychloroquine were significantly associated with poorer prognoses. The prognosis of the subjects receiving calcifediol alone was always better than that for azithromycin and hydroxychloroquine alone or combined.

## 4. Discussion

This retrospective observational cohort study, which included consecutive patients hospitalized for COVID-19, interstitial pneumonia and hypoxia requiring oxygen therapy and treated with high-dose calcifediol and/or corticosteroids, allowed us to evaluate the effect of single and combined use on the need for ICU admission, risk of death and/or poor prognosis. Treatment with calcifediol administered alone improved the evolution of COVID-19, reducing the need for ICU treatment and poor prognosis, confirming in real life throughout the pandemic the results obtained in the pilot study of calcifediol [14]. These results are similar to those obtained in observational studies when calcifediol was administered at high doses [15,16,27] and is consistent with the effects of the VDES on most tissues, including lung and immune cells [13,28]. However, when calcifediol has been used at strictly replacement doses, the clinical response is more limited [29].

There have been many observational studies reporting an association between a deficient 25OHD status and increased susceptibility to infection [30], severity and mortality from COVID-19 [31,32]. However, reverse causality cannot be ruled out [33]. Indeed, inflammation through various mechanisms may be a significant causal factor in the decreased 25OHD serum levels reported in patients with COVID-19 [34].

The VDES is active in all targets of damage by COVID-19, such as the lung and gastrointestinal, epithelium vascular and immune cells. These cells express both 1α-hydroxylase and the VDR, allowing these cells to locally activate 25OHD into 1,25(OH)2D and regulate a large number of their genes [28,32]. If the availability of 25OHD is decreased, whatever the causal mechanism, the well-established protective functions of the VDES against COVID-19 are lost [28,32].

When SARS-CoV-2 replicates, it releases double-stranded RNA that is recognized by Toll-like receptors, members of the pattern recognition receptor family, on respiratory monocytes/macrophages and bronchial epithelial and alveolar cuboidal lining type II cells, in which it induces increased expression of 1α-hydroxylase (CYP27B1) and the VDR to metabolize 25OHD into 1,25(OH)2D. The activation of the VDES/VDR may yield positive effects against COVID-19, enhancing the antiviral response by boosting the production of antimicrobial peptides (AMPs) such as cathelicidin (cAMP/LL37) and β-defensin (DEFB4), as well as modulation–induction of viral autophagy and inhibition of the key Skp2 protein. VDR activation also promotes an antioxidant effect in monocytes by up-regulating glutathione reductase and glutamate–cysteine ligase, which reduces oxygen radical production. In addition, activation of the VDR contributes to maintaining the integrity of the pulmonary epithelial barrier and stimulating its repair [28,32,35].

Moreover, in the adaptive immune system, VDR activation inhibits antigen presentation by dendritic cells, reduces the number of T helper 1 (Th1) cells and promotes the transition to Th2 and T-regulatory (Treg) cells, inducing a shift from an inflammatory to a more tolerogenic status by moderating the intensity of the local and systemic inflammatory immune response from a severe COVID-19 evolution to a more favorable clinical evolution [28,32].

Another potential mechanism of the VDES associated with decreased severity of COVID-19 is its potent negative regulation of the renin–angiotensin–aldosterone system (RAAS) by inhibiting the renin- and angiotensin-converting enzyme 1 (ACE1)/angiotensin II/AT1R cascade and inducing ACE2’s potentiating effect of angiotensin(1–7) on its receptor (MasR), which promotes systemic anti-inflammatory, anti-fibrotic and antioxidant pathways, and reducing vasoconstriction and thrombogenesis, contributing to reducing the severity of ARDS in all its aspects, post-COVID fibrosis [28,32] and probably effects related to long COVID [36].

We use calcifediol as a treatment because it has pharmacokinetic advantages that may give it functional superiority over native vitamin D for use in COVID-19 [28]. It is more hydrophilic and is absorbed after oral intake through the portal venous system and does not require hydroxylation at position 25, which can be hampered during inflammatory processes [37]. Thus, calcifediol is available at high concentrations within a few hours, and in a stable manner, as the substrate for the synthesis of 1,25(OH)2D3 in the kidneys and other target organs in COVID-19 for its endocrine, paracrine and autocrine actions [28,38]. Therefore, the administration of calcifediol enables the biological actions of the VDES before the catabolic mechanisms of the system (e.g., 24-hydroxylase, FGF23 [39], sulphatases [40,41]…) are substantially enhanced. We did not use calcitriol because of its shorter half-life and the increased risk of complications, such as hypercalcemia.

Corticosteroid treatment of our patients did not change ICU admission, prognosis or death risk in moderately sick COVID-19 hospitalized patients. Interestingly, calcifediol-only treatment was found to be more favorable than corticosteroid therapy for all the outcomes evaluated.

Corticosteroids are widely used to treat a variety of medical conditions due to their ability to suppress the immune system and reduce inflammation by inhibiting the expression of multiple proinflammatory cytokines and chemokines, as well as activating different immune cells in a manner that resembles the VDES [17,42]. In contrast to calcifediol, corticosteroids impair the production of antiviral cytokines (IFN I) [43] and their signaling pathways, leading to a decrease in the expression of IFN-stimulated genes [43]. Therefore, if corticosteroids are administered early in the course of a viral infection, they are likely to interfere with and reduce both the efficacy of IFN production and the down-regulation of IFN-stimulated genes [17,42], thereby facilitating viral replication and spread by enhancing the mechanisms of SARS-CoV-2’s action on innate immunity [38]. Corticosteroids also down-regulate the mRNA expression of the antimicrobial peptide genes cAMP and β-DEFB4, lysozymes (LZY) and secretory leukocyte proteinase inhibitor 1 (SLPI) in vitro and in vivo and reduce the expression of the human cathelicidin gene, enhanced by VDR stimulation [44]. Corticosteroids up-regulate multiple components of the renin–angiotensin–aldosterone system (RAAS), down-regulating angiotensin-converting enzyme 2 (ACE2) [45]. Therefore, corticosteroids are effective in reducing the maladaptive hyperinflammatory response, but by decreasing innate immunity, they can enhance the immune-evasive effect of SARS-CoV-2, which is particularly serious in more susceptible patients, decreasing viral clearance [21,23] and causing the inflammatory hyper-response characteristic of acute respiratory distress syndrome (ARDS) associated with COVID-19 [38]. This dual mode of action may explain the paradox found in the results of the improvement or worsening of prognosis, and even death, depending on the time of disease, progression and the severity of the disease [46]. Calcifediol is a treatment whose antiviral action reduces the reactive hyperinflammatory response and which can be combated early in the course of the disease [38,46] at low cost in a formulation not available in the USA, the UK, Australia or much of Europe [37].

One strength of the present study is its similarity to a large, high-powered observational macro study involving 26,508 veterans reviewing the interaction of “vitamin D” (a designation, which included vitamins D2 and D3 and calcitriol and corticosteroids in a mortality analysis, also during the early waves of virus infection, prior to the start of vaccine administration. In hospitalized patients with PCR-confirmed COVID-19, the results on the use of native vitamin D and/or calcitriol resemble our results. The use of “vitamin D” in any form alone or in association with corticosteroids decreased the risk of death relative to the use of corticosteroids alone [47].

The major limitation of our study is its observational nature during three episodes of the COVID-19 outbreaks before the widespread availability of vaccines. However, the known risk factors for the severity of the disease were not statistically different between the different treatment arms of our study, except for BMI. In fact, patients treated with calcifediol only were more frequently obese in comparison with the other groups and nevertheless had the best outcome.

In summary, this retrospective observational cohort study, which included consecutive patients hospitalized for COVID-19 and treated with high-dose calcifediol and/or corticosteroids, according to the clinical practice guidelines, allows us to evaluate the effect of each single and combined drug use on the need for ICU admission, risk of death and/or poor prognosis. Patients treated with calcifediol alone required fewer ICU admissions and had a better prognosis than those on corticosteroids, and when calcifediol and corticosteroids were given together, the response was more favorable if calcifediol was given first.

## 5. Conclusions

Therefore, based on all the available evidence, we can conclude that calcifediol, with limitations derived from the study’s observational design, may be the treatment of choice in hospitalized patients with COVID-19, not administering corticosteroids until the natural history of the disease reaches a stage of severe hyperinflammation. Moreover, calcifediol is a cost-effective treatment without major adverse effects, which could have positive implications for the treatment of the disease worldwide [48].

## Figures and Tables

**Table 1:** Population demographics.

		Treatment					
Variable		Calcifediol *n* = 68	Corticosteroids *n* = 112	Both *n* = 510	None *n* = 38	Total *n* = 728	*p*
Outbreak	1 2 3	63 1 4	5 94 13	7 254 249	31 7 0	106 356 266	<0.001
Female Male		32 36	46 66	186 324	16 22	280 448	0.319
Age		52.38 ± 10.47	52.74 ± 11.02	52.72 ± 9.99	52.39 ± 12.61	52.68 ± 10.33	0.916

**Table 2:** Personal history by treatment group.

	Treatment				
Variable	Calcifediol *n* = 68	Corticosteroids *n* = 112	Both *n* = 510	None *n* = 38	*p*
Lung disease *	10 (8.13%)	17 (13.82%)	92 (74.80%)	4 (3.25%)	0.564
Renal impairment **	0 (0%)	1 (10%)	8 (80%)	1 (10%)	0.688
Diabetes *	3 (3%)	15 (15%)	76 (76%)	6 (6%)	0.126
High blood pressure *	15 (5.88%)	37 (14.51%)	189 (74.12%)	14 (5.49%)	0.102
Thyroid disease **	8 (14.04%)	9 (15.79%)	38 (66.67%)	2 (3.51%)	0.570
Cardiovascular disease **	4 (6.67%)	7 (11.67%)	46 (76.67%)	3 (5.00%)	0.759
Biological treatment **	0 (0%)	0 (0%)	4 (100%)	0 (0%)	1.00
Immunosuppressive treatment **	7 (26.92%)	2 (7.69%)	17 (65.38%)	0 (0%)	0.026
Cancer **	6 (18.75%)	6 (18.75%)	19 (59.38%)	1 (3.12%)	0.232
Obesity/overweight *	5 (1.27%)	63 (15.99%)	322 (81.73%)	4 (1.02%)	<0.001

The values in parentheses refer to the percentage of patients with the pathology who received this treatment. * chi-square; ** Fisher’s test.

**Table 3:** Analytical variables with significance by treatment group.

	Treatment			
Variable	Calcifediol	Corticosteroids	Both	None
Lymphocytes	1198.82 (501.35)	1021.88 (515.17)	1071.18 (587.43)	1120.79 (498.01)
Eosinophils	20.00 (55.93)	32.00 (24.06%)	35.77 (77.38)	13.89 (32.98)
Procalcitonin	0.73 (4.29)	1.43 (9.24)	0.14 (0.25)	4.57 (27.25)
IL-6	25.78 (67.99)	16.18 (25.21)	23.14 (89.99)	15.03 (13.68)
IO2	345.17 (68.97)	315.40 (80.64)	320.58 (61.65)	352.67 (84.90)

Values expressed as mean ± standard deviation. Abbreviations: IL-6: interleukin 6; IO2: oxygenation index.

**Table 4:** Drugs administered and number of patients who received them by treatment group.

	Treatment				
Drug	Calcifediol *n* = 68	Corticoids *n*= 112	Both *n* = 510	None *n* = 38	*p*
Hydroxychloroquine *	59	6	10	27	<0.001
Azithromycin *	60	102	336	30	<0.001
Ceftriaxone *	55	100	362	31	<0.001
Calcifediol *	68	0	510	0	<0.001
Glucocorticoids *	0	112	510	0	<0.001
Lopinavir / Ritonavir **	7	3	0	13	<0.001
Interferon **	2	1	1	11	<0.001
Tocilizumab **	5	7	83	0	<0.001
Adalimumab **	0	5	16	0	0.273
Immunoglobulins **	0	3	1	1	<0.05
Bevacizumab **	0	1	0	0	0.299
Antithrombin **	1	7	66	0	<0.001
Sarilumab **	0	0	7	0	0.686

* chi-square; ** Fisher’s test.

## Data Availability

Some or all of the datasets generated during and/or analyzed during the current study are not publicly available due to privacy but are available from the corresponding author on reasonable request.
